# Mr. H. Sandwith on the Epidemic Fever of Bridlington and Vicinity

**Published:** 1821-06-01

**Authors:** 


*A History of the Epidemic Fever, which prevailed in Bridlington and the Neighbourhood, in the Years* 1818 *and* 1819. By HUMPHRY SANDWITH, Surgeon, Bridlington. *Also, Observations in Medicine and Surgery.* By THOMAS SANDWITH, Surgeon, Beverley. One volume, 8vo, pp. 327. London, 1821.

---

"Leges omnium salutem singulorum saluti anteponunt; sic vir bonus et sapiens, et legibus parens, et civilis officii non ignarus, utilitati hominum; plusquam unius alicujus, aut suæ consulit." *Cicero de fin. bon. et mal.*

---

SYDENHAM, in his epistle to Dr. Brady, complains that four years after the publication of his Observations on the Use of Bark in Intermittents, his precepts remained unnoticed, or contemned by his cotemporaries. Nay, in return for his exertions to enlighten his professional brethren, the ENGLISH HIPPOCRATES was loaded with vituperations by those persons—"de quibus hoc habeo dicendum," says he, "quod si vita innocua hominis, qui neminem neque facto, neque dicto, læserat, me ab illis sartum tectum reddere valuisset, nunquam in me detonuerant." Thanks to the PRESS, no valuable work can now slumber unnoticed for four long years—or even as many months. Neither dare Slander raise its snaky heads against the promulgators of useful facts or ingenious doctrines, without danger of a fatal recoil on itself. The muzzled monster can now only gnaw, in dark holes and lurking places, the reputations of those whom it dare not openly assail; because public sentiment will not permit such unprincipled aggressions in matters of science —and particularly medical science. We maintain then, that in these respects, we live in a better age than did Harvey or Sydenham. In ancient times FAME marched on foot, "magnas *it* fama per urbes," however high she might lift her head among the clouds; but now Fame and Knowledge are wafted with more than was ever fabled of Cyllenian velocity over every portion of the civilized globe. That the press pours forth error with truth, like fabled FAME of old, "tam *ficti* pravique tenax, quam nuntia *veri*," is not to be denied, nor even wondered at, considering the imperfections of our nature, the narrow limits of our capacities, and the infancy of our science; yet, were the proportion ten times greater than it is, it would weigh as nothing in the light of an objection to books. The paramount utility of reading does not consist in the quantum of information directly received, though that is considerable; but in the mental exercise and excitement thus produced, by which the intellectual faculties are strengthened and rendered more capable of profiting by direct and personal observation and experience. Books therefore which are well calculated to stir up *thought* in the reader, are often of more service than those which contain richer materials buried under an awkward exterior, and conveyed in confused or barbarous language. The Pedagogues who clothed the ideas of Sydenham in such difficult and elaborate language, (a curse in perpetuity on candidates for academic honours, per omnia secula seculorum,) must have done him a great injury in his own days, and rendered every subsequent translation participant in the deformities of the original. Medical writers are now growing wiser, and many of the wisest attend to language and style. Those who neglect the attractions of composition, and particularly those who, from carelessness or innate defect, convey their thoughts in obscure instead of luminous diction, suffer from a cause which, though not openly assigned by their readers, is not the less operative in its results. It will not do, in these days, to spread our thoughts on paper in characters that require deciphering—or in long sentences that demand reperusal before their drift can be understood. Yet there are, and have been, many writers whose obliquity of intellect (for we know not how otherwise to account for the phenomenon) renders turbid every stream of thought that passes through their brain, and thus they mystify that science which they undertake to elucidate. These observations are general, and not addressed to particulars—still less are they applicable to the work before us, which evinces a vigorous conception, united with perspicuous delivery, in its intelligent authors.

The first division of the work, (on the epidemic fever,) by Mr. Humphry Sandwith, is the only part which we shall attempt to analytically delineate in the present number of the Journal, reserving the miscellaneous observations, by Mr. Thomas Sandwith, for our next. To say the truth, we have been much gratified by the perusal of this volume generally, and even where we cannot entirely coincide in the views of Mr. H. Sandwith, we admire his ingenuity, his candour, and his independence of mind. The strength of his judgment too, and the force of his reasonings, are well calculated to make strong impressions in his favour on the minds of his readers.

Messrs. S. address their work to Dr. John Simpson, of Malton, as illustrative of principles long acted on with success by that Physician, while they thus publicly acknowledge their professional obligations for the benefit of his conversations and correspondence. We are always gratified to observe this unanimity and friendly cooperation among the members of our profession; and deprecate most heartily every act, word, and writing, calculated to produce irritation, excite the turbulent passions, or keep up those repulsive manners which too often, alas! unhinge the frame of medical society, by sowing the seeds of discord where harmony should prevail. Every wise and good man should discountenance, publicly and privately, all tendencies of this kind, as inimical to peace of mind among individuals, and subversive of that public esteem which can only be maintained by the profession, as a body, while philosophic decorum and mutual urbanity prevail.

The first section of the work before us is dedicated to meteorological observations, and a topographical sketch of Bridlington and its vicinity, which we shall pass over; the second section is headed—"Internal Evidences" of the epidemic, comprehending, 1st, those cases in which the nervous system suffered merely from the agency of general fever—2dly, those in which the nervous system was thrown into additional disorder by intensely sympathising with inflammation or congestion of some important organ—3dly, where the nervous system appeared to be implicated in idiopathic or primary inflammation or congestion.

I. *First Species, or Simple Typhus.* Mr. H. Sandwith justly observes, that when we come to class fevers, we find cases holding a place so exactly intermediate, that it is difficult to assign them their situation. Thus, what he denominates "simple typhus, bordering on the inflammatory," might, he conceives, be considered with much justice, as mild cases of the inflammatory species; yet, the description of this variety forms a sort of connecting link which unites simple and inflammatory typhus.

During the late epidemic, thirty-four examples of simple typhus presented themselves to our author, of which, eleven assumed its more aggravated character. To the readers of this Journal the symptoms of simple typhus need not be detailed. They are slightly sketched by our author, from a conviction that a minute detail was unnecessary. Although this form of fever had an open expression, yet the utmost sagacity could not, from early symptoms, predict, with certainty, that the disease would pursue a safe and gentle course.

"In fact, many examples of this species of fever, I allude to those of 'Simple Typhus bordering on the Inflammatory,' were restrained within the limits of the present classification, and prevented from passing into the inflammatory or congestive forms by the timely use of the lancet or leeches, in other words, *by anticipating evil.*" P. 17.

Our author concludes, with Dr. Percival, that the degeneracy of this fever was considerably, if not principally, dependent on delay or neglect of energetic remedial treatment.

*Simple Typhus bordering on the Inflammatory.* The aspect of this variety impresses the medical attendant with the sense of more existing danger than in the preceding. In our author's experience there was greater prostration of strength—higher degree of temperature—greater local pain, whether in the head, chest, or abdomen. Where pulmonic disturbance prevailed, the distress was more exquisite. The bowels were more intractable—nervous irritation, both mental and corporeal, more distressing.

"The nature of simple typhus will be further illustrated by a view of its duration, allowing for the influence of treatment. Premising that critical days were seldom noticeable, I find there were three recoveries within the first week, seven in a fortnight or less, seven in three weeks or less, eleven in a month or less, four in five weeks or less, and two in about six weeks." 20.

Mr. S. seldom marked the regularly formed crisis so well described by Dr. Cheyne, as consisting of three stages—one of general disturbance, a second of rigor, and a third of perspiration, resolving the complaint. On the contrary, a gradual though increasing perspiration, or an improving and soluble state of the bowels, or long-continued and refreshing sleep, with the gradual resumption of impaired vital functions, terminated the disease.

II. *Second Species,* where the nervous system intensely sympathizes with inflamed or congested organs. In the first species no lives were lost—in this, some few failures took place. The author confesses (perhaps with more candour than justice) his conviction that more energetic measures might possibly have saved even these. But men will die, under the most judicious treatment sometimes. When the French soldiers at Flushing petitioned Napoleon to remove them from a situation where they were dying, his answer was equally laconic and true—" L'homme meurt par tout"—" men die in all kinds of places."

The nervous system is disturbed by simple inflammation of an internal viscus, as well as in simple typhus. This disturbance will, of course, be much increased when both morbid conditions are combined. In this variety, the typhoid countenance was rendered peculiarly impressive by the physiognomy of distress or even agony. Intellect, sensation, the development of temperature, and the motion of the voluntary as well as involuntary organs, were all seriously affected. Acute bodily pain, no less than occasional injury of the different organs of sense, indicated much interference with the function of sensation. The secreting organs were much deranged—the pulse ranged from 100 to 140, and the animal heat was morbidly increased, in half the cases, excessive.

Mr. S. thinks that one great cause of the nervous disturbance may be attributed to the pulmonary affection, which, by obstructing the free return of blood from the head, deranges the sensorial functions. "But it is not improbable, that the delirium may also, in part, depend on those inexplicable sympathies which exist in the nervous system." In some cases Mr. Sandwith found that the brain became inflamed after sympathizing for some time with other inflamed organs. The duration of this species varied exceedingly—recovery taking place at different periods from the 5th till the 43d day. The occurrence of critical days was noticeable only in two instances. Of the two fatal cases, one died on the 17th, and the other on the 18th day.

III. In the THIRD SPECIES Mr. S. conceives the nervous system to be essentially implicated in an idiopathic and primary inflammation—or congestion, with occasional subsequent disturbances elsewhere.

In the Bridlington epidemic the numbers of this formidable variety nearly equalled those of both the preceding. In order to prove " that the disorders *observable* in the nervous system have their *origin* in vascular derangement," Mr. Sandwith presents us with the minutes of a dissection—the only dissection, unfortunately, which he was allowed to make. The symptoms of this case nearly corresponded with the account given by Dr. Armstrong of the less severe forms of congestive typhus. The fever lasted three weeks, ending on the 21st day.

" I only witnessed its course during the last eleven days; in the first five of which there was generally slight yet distinct mental aberration, with occasional maniacal extravagancies; and in the last six almost constant delirium, with great reduction of the bodily strength.

Every symptom pointed to the centre of the nervous system, as the vitiated source of the febrile phenomena. The duration of the disease, and the peculiarities of the patient, withheld my hand from unsheathing the lancet. She was a delicate female, had borne many children, and gave suck at the time; she was moreover the subject of domestic affliction, and from extreme poverty was ill-nourished. The appearances of debility, though more seeming than real, served still further to intimidate me. On these accounts, leeches, purgatives, alteratives, and blisters, with a seton in the neck, were relied on only to prove their futility. The case occurred in September, 1818; the patient's age about forty; the weather cold and moist. In an adjoining street another fever patient died, I believe, with most of the symptoms described in this case. In that case too no blood had been abstracted. My patient had long been the subject of occasional paroxysms of low melancholy derangement." 34.

On dissection the dura mater was found strongly adherent to the cranium—the pia mater morbidly attached to the dura mater, in certain places—great effusion of semi-transparent fluid under the pia mater—effusion into the ventricles and theca vertebralis, as far as the eye could reach—the pia mater bloodshot in the extreme between the convolutions of the brain—points of blood issuing from the sliced surfaces of the cerebrum and cerebellum. In the chest some adhesions were seen between the pleura pulmonalis and pleura costalis, the thoracic viscera themselves being sound. In the abdomen the principal morbid appearance was a vascular turgidity in a portion of the ileum.

That the above appearances, and "the disorders *observable* in the nervous system," resulted from derangement of the vascular system, no one will deny; but we do not see how it can be inferred from this that the *original* or primary derangement of function in the nervous system was produced by the vascular system. On the contrary, all the physiological and pathological phenomena appear to lead us to the conclusion, that the first morbid impression is made on the nervous system—that vascular derangement follows, and that alterations of structure is the result of all.

The observations which follow from page 36 to page 65 we must pass over, as incapable of analysis, and being interspersed with numerous quotations from the writings of Drs. Armstrong, Clutterbuck, Percival, and Bateman.

The third section of the work embraces the important subject of Treatment, in which our author finds it impossible to avoid taking an extensive survey of the different therapeutics employed in typhus fever. The principal of these is blood-letting; the utility of which, Mr. S. considers as established by the experience of two centuries.

"They who can view without concern the progress of the vascular derangements which are apt to occur in fever, as chiefly indicated by the most obvious deviations in the economy of the nervous system, and see this system itself exposed to the risk of irreparable organic lesion with the same indifference, cannot habitually connect symptomatology with morbid anatomy; neither can they reflect on the strength of those sympathies, and the force of those mutual relations, whose unimpeded harmonious play is essential to life in man and the higher order of animals. Their reliance on the powers of nature unaided, or assisted only by the most contemptible auxiliaries, to defy a coalition of hostile influences against existence itself, makes not only the depth of their pathological researches questionable, but their acquaintance with physiology itself liable to suspicion." 68.

The epidemic witnessed by our author was not to be trifled with. Where blood-letting was not employed at all, or tardily and inefficiently had recourse to, organic lesions were the consequence. When, in bad cases, our author substituted leeches for the lancet, "disappointment and vexation awaited him."

From page 77 to page 107, our author undertakes to prove "that blood-letting is by far the most efficient agent ever wielded in the contest with fever." The general truth of this proposition is proved, he thinks, by the fact, that the mortality of continued fever is much less now than formerly. But unless he can shew that fevers are the same now as formerly, and that the other items in the treatment and regimen are the same, we fear his proposition must appear extremely defective. For our own parts, we are firmly convinced that no two epidemics are alike, or will yield to precisely the same treatment—a belief as old as the days of Sydenham at least. At the same time we believe, with Mr. Welsh, that in *all* fevers, blood-letting is our principal remedy to guard against or remove inflammatory affections of particular organs arising in the course of the fever.

"It may confidently be asserted, that in all severe cases of the disease in which recovery takes place without bleeding, that event may be considered *an escape* rather than *a cure*. The distinction may seem an invidious one to the abettor of the doctrines which it is my aim to refute. Nevertheless it is sanctioned by a reference to the pathology of fever, no less than by the plainest dictates of experience. Nothing can more forcibly illustrate my meaning than the following memento by Dr. Bateman. "I lately witnessed the complete and immediate extinction of the fever in two cases, (not in the House of Recovery,) by a single blood-letting: the one performed on the fourth and the other on the fifth day. The head, in both, was considerably affected with threatening delirium; but the pain and intellectual disturbance were instantaneously removed, and the patients left their beds in two days. In one of these patients an attack, marked by precisely the same symptoms, had occurred three years ago, which was not arrested by a free cupping; but the fever went on with severity, and terminated with a formidable derangement of the intellectual functions, and a tedious recovery."* P. 79.

* Bateman on Fever, p. 99.

While we perfectly approve of Dr. Bateman's treatment in the two cases abovementioned, we believe that very few of our brethren, who have seen much of typhus fever, will be disposed to admit that such fever is often to be cut off on the fourth or fifth day of its march. An inflammatory affection of the encephalon will, of course, present all the phenomena of fever, and may be sometimes arrested in the above manner; but when fever rises in consequence of typhous infection, we have good reason to know that it will rarely yield to the treatment in question, till it has run a course.

In nothing, Mr. S. observes, was the power of the lancet more conspicuous than in the rapid removal of delirium, notwithstanding the deep hold which that affection had taken on the sensorium, "and though acute functional disturbance had nearly lapsed into fatal organic lesion." 80. "Used still later, and when incipient effusion perhaps, or approaching gangrene, has sapped the foundations of life, the lancet will only accelerate death." On such melancholy occasions our author has pushed the cordial plan to the fullest extent, without any corresponding advantage. In a few most desperate cases, (of a former epidemic,) our author has succeeded, "but which, it appears to him, were examples of general exhaustion merely, accompanied perhaps with slight local inflammatory congestions." He suggests, with Dr. Wilson Philip, in such cases, the use of galvanism.

Cases of extreme exhaustion, according to our author's observations, were by no means peculiarly the result of the evacuant practice. They almost exclusively occurred "under a plan of treatment which excluded the lancet."

"And a very good pathological reason can be assigned for the fact. The truth is, that by those local congestions which are the cause of endlessly repeated reaction, the duration of fever is incalculably augmented, and the powers of life exhausted. On the other hand blood-letting has evidently the power of abridging the duration of fever. 'Instances of this fact,' says Dr. Welsh, 'might have been multiplied to an immense extent, almost to half the number of patients received into Queensbury House.'" 85.

To the question "how late can we bleed in typhus fever?" no satisfactory answer can, of course, be given, since, like the quantity to be taken away, it must depend on the existing circumstances of the case. The cases published by Dr. Welsh prove that we may bleed at a much later period of the disease than was generally imagined, though the extent to which that gentlemen carried the measure lies open to some objections.

The next point which Mr. Sandwith urges is, that blood-letting is an indispensable measure of *precautionary* safety in the majority of cases. After several acute remarks, and some strictures on recent publications, our author makes the following statement of his own:—

"Upon the whole, therefore, the rule of bleeding in every case, when we are called in early, in order to avoid the unpleasant dilemma of being surprized by unforeseen danger, seems to me generally, though not universally applicable. We have instanced the exception of peculiarity of type. We might further name, as exceptions to the practice, extreme debility, and a peculiarity of constitution not bearing general abstractions of blood. In the practice of medicine, indeed, nothing is more annoying than the union of congestion with constitutional debility; as for instance, where in a state of health we find cold hands, a weak and hurried action of the arteries, frequent perspirations, weak digestion, and great nervous irritation.* The circumstances which would forbid the use of general bleeding, with a view to anticipate danger in fever, are not therefore very numerous. In the latter part of my practice, I have employed this method with scarcely any exception, and always with the happiest effects. In several of them, though the fever went on with considerable severity for some time, we were never appalled by the development of a single untoward symptom; though from the violence of the attack in some instances, great danger would in all probability have succeeded to less active measures. Sometimes delirium never appeared at all; at other times, it was so slight as scarcely to deserve the name. In one case, bleeding on the second day 'arrested the fever' instantaneously. The same happy consequence was observed in a second instance also. But it is imperiously necessary that the bleeding should be a free and decisive one, and that in urgent cases it should be repeated with a vigour proportioned to circumstances; otherwise it will be more mischievous than salutary, for it may, if deficient, conceal increasing danger under an imposing but deceitful appearance of improvement.'' 104.

With respect to the subject of anticipating danger in fever by general bleeding in the earliest periods of reaction, "it cannot excite our surprize that we should meet with so little information in the works of our best hospital writers on fever." But if Mr. Sandwith had consulted the writings of army and navy practitioners, he would have found much information on this point. We refer him, therefore to the writings of Jackson, Irvine, Boyle, Dickson, M'Arthur, Burnett, Fergusson, Sheppard, and many others who, in watching the health of our soldiers and sailors, had the means of employing the most active measures in the earliest stages of fevers. Mr. Sandwith's observation can apply only to the physicians of civil hospi-

---

"* And yet great stress cannot be laid even on this exception: for the most experienced physicians have remarked, that there are cases of constitutional idiosyncrasy, where, under ordinary circumstances, the loss of blood is so ill borne, that a very few ounces taken from a vein, or even by a leech, is followed by syncope and by excessive languor. and other ill consequences, for days and weeks afterwards; and yet the same patient under an attack of febrile symptoms, bearing even the slightest marks of inflammation, shall suffer a moderate bleeding without inconvenience, and even repeatedly during the progress of the disease with manifest advantage. For this remark I am indebted to Dr. Storer of Nottingham." P. 103.

tals, where, of course, disease is but seldom seen in its nascent movements.

"I have not much to offer on the virtues of other remedies in fever, besides venesection. I found mercury a valuable auxiliary to the lancet, though I always premised adequate depletion by the latter. I employed it in the inflammatory and congestive forms of the disease for the same purpose, and with the same effects, as when given to suspend the adhesive stage of simple inflammations of the viscera. Under these circumstances, and after due depletion, I saw the most beneficial results from its cautious employment. I preferred the pil. hydrargyri, in moderate doses, so as not to pass off by the bowels, in conjunction with digitalis, to calomel in union with opium, as recommended by Dr. Armstrong." 108.

In some protracted cases with restlessness and nervous irritation, moderate doses of laudanum and antimonial wine, procured sleep and hastened recovery. "Friction of the abdomen with an anodyne liniment, preceded by fomentations, was useful at a late stage, in conjunction with mild aperients, in removing pain unaccompanied with tenderness on pressure."

"In the early periods of the disease, I employed saline cathartics, and occasionally calomel, with a liberal and steady hand, taking especial care, however, to avoid hypercatharsis. As the debility increased, I either combined camphor with senna mixture without salts, or substituted castor oil or rhubarb. If any measure can be substituted for bleeding, it is free purgation, which in case (12) invariably averted a strong tendency to pulmonary organic mischief unaided either by the lancet or leeches." 109.

Mr. S. informs us that experience soon convinced him that it was an illusion to expect a collapse after the first week of fever in all ordinary cases. Accordingly he latterly delayed cordials, in general, till about three weeks had elapsed, and then allowed wine in the most sparing quantities only. Given at an early period, our author justly observes, cordials augment debility by increasing fever and favouring local determination. Similar restrictions apply to diet.

Our author denies the validity of the common opinion that this disease is very simple as it occurs in children.

"The record of the Bridlington Epidemic is of value, as shewing that childhood is by no means an invariable protection from its most malignant attacks. In fact one of its most prominent features was the severity of the disease in children and young subjects; who will be found to have constituted a majority in the list of fatal cases. The young practitioner will thus be put on his guard not to relax from that close observation necessary to enable him to detect the earliest development of danger. Many excellent authorities also advise milder measures for children than for adults. Thus Dr. Prichard, in his History of the Bristol Epidemic, observes, 'In persons of weak habits or much exhausted, and *in children under ten*

*years of age,* instead of bleeding from the arm or temporal artery, leeches were applied to the head.' The rule, as applicable to the eruptive fevers, has been ably controverted by Dr. Armstrong, in his invaluable remarks on the treatment of measles. And as it applies to typhus fever, my own experience warrants no such distinction. A reference to the Tables will shew, that in several instances leeches were wholly inefficacious; while the lancet was signally successful in others of equal danger." 111.

It was our intention to have fully noticed Mr. Sandwith's concluding section on "the nature of fever," which contains many ingenious observations and sound principles of pathology and practice, but we were deterred, when we looked at the long list of works still waiting for analytical review. The same reasons prevent our dwelling on some interesting papers contained in the appendices to this portion of Mr. Sandwith's work. We cannot, however, pass them over entirely.

The first paper is from Mr. Thomas Sandwith, containing recollections of the epidemic, in which he observes that the writings of Sydenham, Rush, Jackson, Beddoes, Clutterbuck, and Hamilton, had in some measure prepared him against "the theories of Brown and Cullen, and the ingenious trifling of Currie." He was determined therefore never to prescribe for the *name* of a disease, but combat the symptoms, whatever they might be—a salutary rule of conduct he learnt from Rush.

The first case that occurred to him made a strong impression on his mind. The patient had been nearly a fortnight ill with fever, and thinking the lancet was inapplicable at that late period, he had recourse to leeches and purgatives, which procured a temporary amelioration of the symptoms.

"At the end of the third week, however, instead of crisis, an universal trembling of the limbs and subsultus came on, and the patient died. The event of this case opened my eyes to the necessity of using the lancet, even in the advanced stages of fever; an important addition to my knowledge." 155.

After relating a number of interesting cases, Mr. T. Sandwith lays down the following rule, in which we cannot entirely coincide.

"We should never trust to mild measures in typhus, when the disease sets in mildly. So certain is the occurrence of inflammation in the course of typhus, if not prevented by early bleeding, that the cases in which it does not appear are but exceptions. The objections against blood-letting seldom apply to its early use; and if the fever is mild, it will make it milder." 162.

Upon the whole Mr. T. S. thinks that "the modern treatment of fever is about as successful as that of inflammation." He treated 103 cases of the epidemic, of which six died.

"I cannot conclude these recollections better than in the words of an eminent and honest physician, who flourished a century ago.

Discoursing with Dr. White, whose papers he had perused, on a larger phlebotomy in fevers, 'I have,' says he, 'been long ago convinced that a considerably larger phlebotomy than in practice would be much better many times, than whole gallipots full of medicines for the patient.' N. B. You will find the practices of the moderns in fever anticipated by the sagacity and courage of Dr. White. If you have not read his book, read it.

"Vixere fortes ante Agamemnona
"Multi." 165.

The second appendix contains a very sensible communication from Mr. Dunn, surgeon, of Pickering, now of Scarbro. He informs Mr. S. that, "where bleeding was used on the first three days, he knew of no fatal case, and even when resorted to in the most hopeless cases, he means in those where the febrile action had become rooted in the system, he always found it to tranquillize and cool the patient above all other measures." Mr. Dunn almost lost his confidence in leeches, though in Scarbro, where the vascular excitement does not run so high as in the country, he generally finds them sufficient. He prefers, in general, the lancet.

"With regard to your question of particular organs being affected, I must answer, that here the greatest variety prevailed. The lungs were often attacked in the later periods, but occasionally even in the earlier of the disease; from thence the pain and affliction would shift to the head, the bowels, the back, the limbs, and vice versa. One day I had to bleed the patient for tenderness of the abdomen, the next for violent pain in the head, and in the third perhaps, or fifth for the uneasiness of the limbs or back." 169.

Children were "amazingly relieved from acute suffering by the lancet and calomel."

"I once gave twenty grains of calomel to a boy divided in three doses before I could procure stools. The boy had been given up by his former attendant. When I arrived he was insensible, labouring under continued stupor; and seemed in a hopeless condition; as soon as the calomel operated he began to recover."* 170.

---

* Mr. Dunn seldom prescribed less than ten grains of calomel, and even larger doses, in adults. An interested cry has been raised by some modern practitioners against the occasional exhibition of large doses of calomel in certain dangerous diseases, especially in the hotter climates of the earth, where disorganization quickly supervenes on inflammation, if not promptly counteracted. A *scruple dose* of calomel has therefore been looked upon with horror, as a thing unprecedented and unwarrantable. If these squeamish practitioners will turn to Sydenham's epistle to Dr. Patman, they will find that the English Hippocrates exhibited this medicine in similar doses, without any *scruples* at all.

"Vel bis in Septimana pil. sequentes exhiberi poterint.

℞. pil. ex. duobus ℥ss. *mercurii dulcis* ℈j. cum Q. s. opobalsami f. pil. iv. sumendæ summo mane."

Now if Sydenham gave the above dose twice a week in a *chronic* dis-

Mr. Dunn never prescribed the bark as a tonic; in the febrile state he preferred the muriatic acid, and in the convalescent stages, when the lungs had been affected, he used the aqueous extract of myrrh. In Scarbro he has not found it necessary to adopt such bold measures as in the country. He finds a single bleeding at the commencement, with a cathartic and a suitable regimen generally sufficient.

The 3d appendix is a short communication from Mr. W. Hardcastle, respecting the epidemic as it appeared at Newcastle. Mr. H. refers to the account published by Dr. M'Whirter in the Edinburgh Journal, and avers that, as far as his own observations extended, he saw no cases of what might be termed typhus gravior.

"I almost invariably found the fever accompanied with more or less of inflammatory action in the brain or chest; in some few a tenderness in the region of the liver: affections of an inflammatory nature in the viscera of the abdomen (the liver excepted) were but rare at the onset of the complaint." 173.

To this part of the work are appended extensive tables of individual cases exhibiting at one view the sex, age, rank in life, and place, form of fever, date of seizure, and if first visit, predominant symptoms, organ most affected, ordinary treatment, date and mode of bleeding, date of evident decline, date of convalescence, number of days sick, probable cause—general remarks. This plan we think an excellent one, especially in epidemics, where the details of a number of cases would swell out a work, and deter men from perusal.

Besides impressing us with a very favourable opinion of the talents of the authors immediately concerned in the volume before us, its contents have added to our conviction that medical knowledge is attaining an immense diffusion and extent through the remotest ramifications of the profession. Works are now every day emanating from the class of general practitioners, which, a few years ago, would have been exceedingly creditable to the class of physicians. This extension of information must be productive of the most important benefits to the cause of humanity in general, and will, we trust, rapidly raise the professors of our art in their own and in public estimation.

---

ease, we ask where is the dreadful crime of exhibiting the same in acute diseases where the intestinal canal is often so torpid as to be with the greatest difficulty excited to its proper function?

